# Implementation interventions to improve the management of non-specific low back pain: a systematic review

**DOI:** 10.1186/s12891-016-1110-z

**Published:** 2016-06-10

**Authors:** Simon Alexander Mesner, Nadine E. Foster, Simon David French

**Affiliations:** 502, Caspian, Apartments, 5, Salton Square, London, England E14 7GJ; NIHR Musculoskeletal Health in Primary Care, Arthritis Research UK Primary Care Centre, Research Institute of Primary Care and Health Sciences, Keele University, Keele, Staffordshire England ST5 5BG; Canadian Chiropractic Research Foundation Professorship in Rehabilitation Therapy, School of Rehabilitation Therapy, Faculty of Health Sciences, Queen’s University, Kingston, Ontario, Canada; Senior Research Fellow, Centre for Health, Exercise and Sports Medicine, School of Health Sciences, The University of Melbourne, Melbourne, VIC Australia

**Keywords:** Non-specific low back pain, Implementation, Best practice guidelines

## Abstract

**Background:**

Recommendations in clinical practice guidelines for non-specific low back pain (NSLBP) are not necessarily translated into practice. Multiple studies have investigated different interventions to implement best evidence into clinical practice yet no synthesis of these studies has been carried out to date.

The aim of this study was to systematically review available studies to determine whether implementation interventions in this field have been effective and to identify which strategies have been most successful in changing healthcare practitioner behaviours and improving patient outcomes.

**Methods:**

A systematic review was undertaken, searching electronic databases until end of December 2012 plus hand searching, writing to key authors and using prior knowledge of the field to identify papers. Included studies evaluated an implementation intervention to improve the management of NSLBP in clinical practice, measured key outcomes regarding change in practitioner behaviour and/or patient outcomes and subjected their data to statistical analysis. The Cochrane Effective Practice and Organisation of Care (EPOC) recommendations about systematic review conduct were followed. Study inclusion, data extraction and study risk of bias assessments were conducted independently by two review authors.

**Results:**

Of 7654 potentially eligible citations, 17 papers reporting on 14 studies were included. Risk of bias of included studies was highly variable with 7 of 17 papers rated at high risk. Single intervention or one-off implementation efforts were consistently ineffective in changing clinical practice. Increasing the frequency and duration of implementation interventions led to greater success with those continuously ongoing over time the most successful in improving clinical practice in line with best evidence recommendations.

**Conclusions:**

Single intervention or one-off implementation interventions may seem attractive but are largely unsuccessful in effecting meaningful change in clinical practice for NSLBP. Increasing frequency and duration of implementation interventions seems to lead to greater success and the most successful implementation interventions used consistently sustained strategies.

**Electronic supplementary material:**

The online version of this article (doi:10.1186/s12891-016-1110-z) contains supplementary material, which is available to authorized users.

## Background

### Low back pain

Low back pain (LBP) is a major healthcare problem around the world [1–3], recently ranked as the number one cause of years lived with disability [3]. No geographic region, particular age group or section of society is immune from the effect of this condition with the World Health Organisation (WHO) estimating that nearly everyone will experience LBP sometime during their lives [4]. LBP is also a major economic burden, with an estimated 818,000 disability-adjusted life years lost annually worldwide due to work related LBP [5].

For many people with LBP a specific diagnosis is not possible with an estimated 85 % of cases not attributable to specific serious pathology or nerve root irritation [6–8]; thus most patients with LBP have non-specific low back pain (NSLBP) [9, 10]. Clinical practice guidelines based on the best available evidence have been developed to direct management of NSLBP. The Quebec Task Force performed the first comprehensive review into best available evidence for NSLBP in 1987 [11, 12] and highlighted the absence of high-quality evidence to guide clinical decision-making [13]. Since then there has been a considerable increase in research regarding diagnosis, prognosis and treatment of NSLBP [12]. There has also been an increase in the number of best practice guidelines for the management of NSLBP, with at least 11 countries publishing their own national guidelines by 2001 [14]. However despite these multiple sources of best available evidence to inform healthcare practitioners about the management of patients with NSLBP, research has shown that these recommendations are not routinely translated into everyday clinical practice [15–17] the so called ‘know-do gap’ [18]. The mere production and dissemination of clinical practice guidelines in itself is insufficient to change clinical practice [19]. Evidence-based guidelines need implementation interventions to support their implementation into clinical practice and investigating these interventions has led to a new area of science, that of implementation research [20, 21].

A range of implementation interventions have been studied in the field of NSLBP [22, 23]. These interventions vary with respect to their type, target end user, intensity and frequency and they range from simple techniques like postal dissemination of guidelines or educational reminders [24, 25] to more complex, ongoing interventions aimed at changing the entire practice of healthcare practitioners [26, 27]. Within each intervention there are different methods used to impart the information including passive strategies that place the onus on the individual healthcare practitioner to act on the information contained within the guideline [28], specific behaviour change techniques such as persuasive communication [29], opinion leaders [30] and multifaceted behavioural change strategies targeting not only the healthcare practitioner but the organisational system in which they work [31, 32].

### Why it was important to do this review

There are a growing number of empirical implementation studies that have evaluated interventions to support the implementation of best available evidence into clinical practice for NSLBP. These studies vary in terms of the healthcare practitioner behaviours targeted, the nature and theoretical underpinning of the implementation interventions selected and the success or otherwise of these in changing clinical practice. Given the worldwide impact of NSLBP and that implementation of best practice recommendations within guidelines may improve patient outcomes, it is timely to review the available published evidence on implementation interventions.

## Methods

### Aims and objectives

This systematic review aimed to summarise the empirical literature about interventions aiming to support the implementation of best available evidence into clinical practice for the management of NSLBP.

Specifically this review had the following objectives:

1. Determine whether implementation interventions have been effective in terms of improving healthcare practitioner behaviour or patient outcomes.
2. Identify which implementation interventions have been shown to be more effective than others in changing the clinical behaviours of healthcare practitioners and improving patient outcomes.
3. Summarise the implementation interventions used, the theoretical models behind them and the evidence base supporting them.
4. Critically appraise the quality of research studies in this area.

### Design

A systematic review of the English language published literature was chosen as the most suitable study design to address the aims and objectives. Only English language papers were considered as translation of foreign language papers was beyond the resources of this project. It was anticipated that there would not be sufficient homogenous studies to permit numerical pooling of data in a meta-analysis but rather that the review would provide a narrative synthesis of the current research in this field.

### Study eligibility criteria

Randomised controlled trials (RCT), non-randomised controlled trials, controlled before-after studies and studies with an interrupted time series design were included. This followed the Cochrane Effective Practice and Organisation of Care Group (EPOC) guidance for the appropriate types of studies to include in a systematic review of implementation interventions [33]. As per EPOC recommendations cluster RCTs, non-randomised cluster trials and controlled before-after cluster trials were included only if they had at least two intervention sites and two control sites and interrupted time series studies were included only if they had at least three data points before and three data points after the introduction of the intervention. Included studies also had to have used a quantitative outcome measure and to have subjected the measure to statistical analysis, comparing the outcome from the intervention with that from a control or comparison.

Using the PICO format (Population; Intervention; Comparator and Outcome) advocated by the Centre for Reviews and Dissemination, University of York [34] and the Preferred Reporting Items for Systematic Reviews and Meta-analyses (PRISMA) statement [35], the population was defined as any healthcare practitioner involved in the treatment of NSLBP. The included interventions were implementation interventions designed to improve clinical practice for the management of NSLBP. The comparator was the type of control or comparison group and could include other types of implementation intervention(s), no implementation intervention (‘usual care’) or a before/after comparison. The outcomes were the variables analysed for evidence of change and included any measured healthcare practitioner behaviour change or change in patient outcomes, for example rates of requests for radiographs, adherence to best practice guidelines, lumbar spine surgery rate, pain score or physical function score. Only full peer reviewed published papers were included.

Studies that specifically tested an implementation intervention with patients who had serious or specific types of LBP, such as patients with fracture, radicular pain/nerve root compression, were excluded.

### Search strategy

The search strategy for the electronic databases was developed with expert health librarians. A pilot search was first performed on the MEDLINE and EMBASE electronic databases and then expanded. This expanded search was then performed on AMED, Applied Social Science Index and Abstracts (ASSIA), CINAHL PLUS, Cochrane Central Register of Controlled Trials (Central), DARE, EMBASE, ERIC (Proquest), NARIC REHABDATA Literature Database, PEDRO, Physical Education Index (Proquest), SPORTS Discus and Web of Knowledge/Science. A full list of search terms and how they were combined for MEDLINE can be found in Additional file 1. Searching of the electronic databases was from January1987 to 31^st^ December 2012. 1987 was chosen as the start year as this is when the first comprehensive review into best available evidence for LBP was performed by the Quebec Task Force [11, 12]. All references generated from the electronic databases were then exported to Ref Works, an online bibliographic database management software tool and duplicates were removed. In addition to the above, efforts were made to identify additional studies by contacting experts in the field of LBP research and specifically those previously involved in LBP implementation research. These experts were identified from a list of participants at the Odense International Forum XII: Primary Care Research on Back Pain (16^th^-19^th^ October 2012, Odense, Denmark) and were contacted by email to ascertain whether there were any papers currently in press that might be suitable for inclusion in this review that would be published and available in time for this search. The WHO International Clinical Trials Registry Platform website [36] was also checked to identify any registered appropriate studies under the search terms ‘low back pain’ and ‘implementation’.

All titles and abstracts of studies identified from the above searches were then independently screened by the review lead author and by one of the co-authors to determine potentially eligible studies. These potentially eligible studies were then independently screened by two review authors in full text to determine their final inclusion. Any disagreements on eligibility were settled by discussion and consensus and by involvement of the decision of the other co-author if required. Reference lists of the included papers and two systematic reviews about the implementation of clinical practice guidelines in general [37, 38] were searched for any additional studies.

### Risk of bias assessment of included studies

The EPOC risk of bias tool [39] was used to assess the risk of bias of included studies. The choice to use this tool was based on the following: it was developed specifically for use with implementation research studies attempting to change the practice of healthcare practitioners and organisation of healthcare systems; it is suitable for use with different study designs (RCT; non-randomised controlled trials; controlled before-after studies and interrupted time series studies) and it has been used in other similar, published systematic reviews [40–42]. The EPOC risk of bias tool involves rating whether the study has a low risk, high risk or unclear risk of bias in nine different categories (see Table 5). For details of how the EPOC risk of bias tool was applied please refer to Additional file 2.

### Data extraction

A specific data extraction form was developed for this review based on the EPOC data extraction form [43] and the information required to meet the objectives of this review. The data extraction form collected details of study characteristics such as the healthcare practitioners included and location of the study (clinic/hospital or community), the stated primary and secondary outcome measures such as radiograph request rate and healthcare practitioner adherence to best practice guidelines, details of the implementation intervention such as how information was imparted and how data were collected as well as the study’s risk of bias. The data from all included studies were extracted by the lead author with the two co-authors independently extracting data from half of the included studies each. The results of this process were then double checked by the lead author to make sure that all relevant information had been gathered.

### Data synthesis

The data extracted and the characteristics of the studies were compared and contrasted. The implementation interventions tested and the study results were analysed for any consistent patterns in terms of effectiveness (such as type of implementation intervention, type of healthcare practitioner targeted, frequency or duration of implementation intervention, frequency of implementation strategy). Based on the clearest pattern observed the studies were split into three categories based on the duration and frequency of the implementation intervention used. The interventions and results of each of the studies were then described and compared. The risk of bias for the two groupings of study type as per the EPOC guidance (one group including RCTs, non-randomised controlled trials and controlled before-after studies and the other group interrupted time series studies) were also described and compared. A meta-analysis was not possible due to the heterogeneity of the implementation interventions studied and the outcomes reported.

## Results

### Study flow

**Fig. 1:**
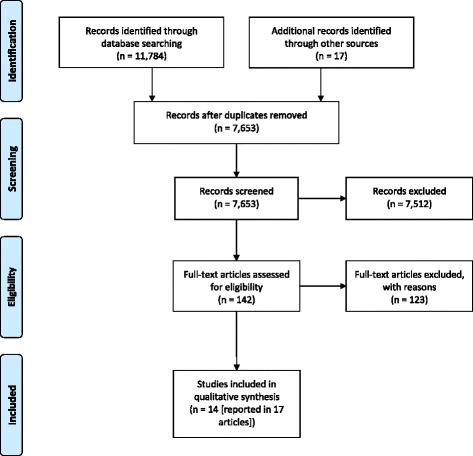
PRISMA flow diagram for included records

Of 11802 potentially relevant citations, 11784 were identified from the database searching and 18 were identified via hand referencing and prior knowledge. Of these 11802 citations, 4148 were excluded as duplicates. After screening the titles and abstracts of the remaining 7654 citations, 7501 citations were excluded leaving a short list of 153 citations. The full text of these were acquired and screened. Of these 153 papers, 136 were excluded. Seventeen papers reporting 14 different studies met the inclusion criteria. Please see Fig. 1 for the ‘PRISMA flow diagram for included records’. Please refer to Additional file 3 for reasons of exclusion for the 136 full text papers.

### Study types

Ten included studies were RCTs and of these, two were cluster RCTs. Two included studies were interrupted time series design, one was a non-randomised controlled trial and one was a controlled before-after study.

### Study outcomes

The included studies examined the effect of the implementation interventions on either healthcare practitioner behaviour change such as adherence to a range of best practice guidelines for treatment of NSLBP or only rates of radiograph requests (10 studies), the effect on patient outcomes such as pain or function (two studies) or a combination (two studies).

### Types of implementation interventions tested

Education programmes were tested in six studies reported in eight papers [23, 26, 27, 30, 31, 44–46]. These programmes utilised a variety of methods to impart the programme content including didactic lectures, follow-up sessions to determine how staff were getting on with the new approach and to answer queries, interactive workshops and printed material. Of these studies, Schectman et al. [23] utilised multifaceted education sessions (an education programme with audit and feedback) for both healthcare practitioners and patients whereas the others focused on education programmes for healthcare practitioners alone. The intervention tested by Becker et al. [44] combined an education programme with training for healthcare practitioners in motivational counselling.

Ongoing reminders and feedback were tested in two studies [24, 28]. The remaining six studies all used different forms of implementation interventions: these were postal dissemination and feedback following audit [22], postal dissemination only [25], educational outreach visits and printed educational material [29], the setting up of special clinics for NSLBP patients [32], feedback on diagnostic test ordering [47] and a special radiograph requisition form [48]. There was no clear pattern of effectiveness in terms of the type of implementation intervention used (see Tables 1, 2, 3 and 4). The same implementation intervention led to significant changes in practice in some studies [31, 44] but not others [27, 46].

**Table 1:** Studies testing a one-off or single event implementation intervention

Study	Intervention and control	Outcome measures	Results		Overall intervention success	
Dey et al., 2004 [29]	IG: Educational outreach visit supported with PEM & access to back pain clinic for non-responders CG: No intervention/usual care	Primary outcome measure(s) not stated.			Only one in five outcomes was statistical significantly different between the IG and CG: Referral to PT or an education programme	
		1. Radiograph request rate	1. 5.1 % IG v 13.7 % CG ρ = 0.62			
		2. Sickness certification rate	2. 17.7 % IG v 19.2 % CG ρ = 0.74			
		3. Prescription of opioids or muscle relaxants (% patients)	3. 18.7 % IG v 18.7 % CG ρ = 0.99			
		4. Referral rate to secondary care	4. 3.4 % IG v 2.3 % CG ρ = 0.12			
		5. Referral rate to PT or an education programme	5. 26 % IG v 13.8 % CG ρ = 0.01*			
Engers et al., 2004 [46]	IG: 2 h educational workshop involving role-play, focusing on the psychosocial model of LBP backed up with PEM CG: No intervention/usual care	Primary outcome measures:	6 weeks:	52 weeks:	6 weeks: No statistically significant differences at 6 weeks in the direction of the IG.	52 weeks: Only one in five outcomes was statistically significantly different at 52 weeks: Consulting an HCP other than GP
		1. Median pain intensity over last 7 days	1. 2 IG v 1 CG ρ = 0.06	1. 1 IG v 1 CG ρ = 0.43		
		2. Functional status: a. Median Roland Morris score	2a. 7.5 IG v 5 CG ρ = 0.06	2a. 2 IG v 2 CG ρ = 0.91		
		2. Functional status: b. Median days not functional in last 6 weeks	2b. 7 IG v 2 CG ρ = 0.03	2b. 0 IG v 0 CG ρ = 0.37		
		3. Median days of work absenteeism in the last 6 weeks	3. 7 IG v 3 CG ρ = 0.01	3. 0 IG v 0 CG ρ = 0.66		
		4. Healthcare use in the previous 6 weeks.	4. 24 % return to GP and 40 % consulting other HCP IG v 17 % & 42 % CG ρ = 0.05 and 0.65	4. 0 % & 36 % IG v 1 % & 50 % CG ρ = 0.24 and 0.01*		
Engers et al., 2005 [27]	IG: 2 h educational workshop involving role-play, focusing on the psychosocial of LBP backed up with PEM CG: No intervention/usual care	Primary outcome measure(s) not stated			Only a lower referral rate to a therapist achieved statistical significance	
		1. Referrals to a therapist	1. 19 patients or 36 % of patients IG v 25 or 76 % CG OR: 0.2; CI: 0.1 to 0.6; ρ ≤ 0.05* but actual value not stated			
		2. Prescription of pain medication on a time-contingent basis	2. 19 or 62 % IG v 10 or 71 % CG OR: 0.7; CI: 0.6 to 6.3; ρ not stated			
		3. Prescription of paracetamol	3. 7 or 23 % IG v 1 or 7 % CG OR: 4.8; CI: 0.1 to 181; ρ not stated			
		4. Prescription of NSAIDs	4. 19 or 61 % IG v 10 or 71 % CG OR and CI not stated; ρ not stated			
		5. Adequacy of patient education rated across nine measures	5. Explained that no specific cause could be detected: 33 or 64 % IG v 22 or 67 % CG OR: 0.9; CI: 0.3 to 2.8; ρ not stated Explained that back pain will ease by itself: 36 or 69 % IG v 23 or 70 % CG OR: 0.9; CI: 0.3 to 3.1; ρ not stated Explained that there is no harm: 44 or 85 % IG v 20 or 61 % CG OR: 3; CI: 0.6 to 16.3; ρ not stated Explained that it is better to accept the pain: 29 or 56 % IG v 17 or 52 % CG OR: 1.7; CI: 0.2 to 13.3; ρ not stated Handed out an information pamphlet: 11 or 21 % IG v 4 or 12 % CG OR: 1.6; CI: 0.4 to 7.8; ρ not stated Advised to stay active: 42 or 81 % IG v 16 or 49 % CG OR: 1.7; CI: 0.3 to 9.0; ρ not stated Advised to gradually increase activity: 45 or 87 % IG v 9 or 58 % CG OR: 3.3; CI: 0.7 to 17.0; ρ not stated Advised which activities to increase when: 11 or 21 % IG v 5 or 16 % CG OR: 2.1; CI: 0.7 to 11.5; ρ not stated Advised to stop activity when in pain: 9 or 17 % IG v 7 or 21 % CG OR: 2.1; CI: 0.7 to 11.5; ρ not stated			
Matowe et al., 2002 [25]	Postal dissemination of guidelines. No CG as the study was a TIS	Primary outcome measure: Lumbar spine radiography request rate.	Mean request rate reduced by 7.7 from 147.8 in the first month with a 0.4 reduction trend over 13 months CI: −24.7 to 40.2; ρ not stated		The reported change did not reach statistical significance.	
Stevenson et al., 2006 [30]	IG: 5-h education session delivered by a local opinion leader CG: Standard in-service session on knee pathologies	Primary outcome measure: Change in PTs’ clinical practice measured using a standardised discharge summary questionnaire assessing time spent using modalities targeted for NSLBP	Number of patients/% of patients with the modality recorded as being used		Only changes in the advice to increase activity levels and attempting to change patient attitudes/beliefs about pain achieved statistical significance. 4 out of 6 primary outcome measures showed no significant difference between groups	
		1. Advice about work situation	1. 42 or 37 % IG v 15 or 35 % CG OR: 1.1; CI: 0.5 to 2.5; ρ not stated			
		2. Advice on return to normal duties	2. 34 or 30 % IG v 13 or 30 % CG OR: 1.1; CI: 0.4 to 3.0; ρ not stated			
		3. Advice to increase activity levels	3. 36 or 32 % IG v 7 or 16 % CG OR: 2.1: CI: 0.7 to 6.8; ρ ≤ 0.05* but actual value not stated			
		4. Encouraging early return to work	4. 5 or 4 % IG v 1 or 2 % CG OR: 1.6; CI: 0.1 to 23.1; ρ not stated			
		5. Encouraging to undertake activities themselves	5. 16 or 14 % IG v 18 or 42 % CG OR: 0.3; CI: 0.1 to 0.9; ρ not stated			
		6. Attempting to change patient attitudes/beliefs about pain	6. 25 or 22 % IG v 4 or 9 % CG OR: 2.6; CI: 0.7 to 9.5; ρ ≤ 0.05* but actual value not stated			

*Abbreviations*: *IG* intervention group, *CG* control Group, *CI* confidence interval, *HCP* healthcare practitioner, *NSAIDs* non-steroidal anti-inflammatory drugs, *OR* odds ration, *PEM* printed educational materials, *PT* physiotherapy, *SS* statistical significance, *TIS* time interrupted series

**Table 2:** Studies testing short-term implementation intervention with no on-going implementation effort

Study	Intervention and control	Outcome measures	Results				Overall intervention success
Bekkering et al., 2005 [26]	IG: 2 x 2.5 hour group training sessions involving lecture and role play 4 weeks apart; postal dissemination of the guidelines and printed educational material CG: Postal dissemination of the guideline and printed educational materials only,	No one primary outcome measure was stated. Outcome measures were adherence to the guidelines as measured against five criteria recorded in the patient record:	In numbers of patients and % of patients:				Successful in altering practice as recorded in patient records. Statistically significant differences reported.
		1. Limiting number of treatment sessions	1. 32 or 27% IG v 14 or 13% CG. OR 2.39; CI 1.12 to 5.12; *p* ≤ 0.05* but actual value not stated				
		2. Setting functional goals	2. 188 or 79% IG v 180 or 71% CG. OR 1.99; CI 1.06 to 3.72; *p* ≤ 0.05* but actual value not stated				
		3. Using mainly active interventions	3. 183 or 77% IG v 154 or 60% CG. OR 2.79; CI 1.19 to 6.55; *p* ≤ 0.05* but actual value not stated				
		4. Giving adequate information	4. 229 or 96% IG v 221 or 87% CG. OR 3.5; CI 1.35 to 9.55; *p* ≤ 0.05* but actual value not stated				
		5. Making all 4 above recommendations.	5. 96 or 42% IG v 75 or 30% CG. OR 2.05; CI 1.15 to 3.65; *p* ≤ 0.05* but actual value not stated				
Bekkering et al., 2005 [45]	As Bekkering et al., 2005 [45] above.	Primary outcome measures:	At 6 weeks:	At 12 weeks:	At 26 weeks:	At 52 weeks:	Unsuccessful. There were no statistically significant differences between the groups. ρ values not stated
		1. QBPDS score	1. 24 IG v 23.5 CG	1. 20 IG v 17.5 CG	1. 16 IG v 11 CG	1. 17 IG v 13 CG	
		2. Pain rated on a numerical scale	2. 3 IG v 3 CG	2. 2 IG v 2 CG	2. 2 IG v 1 CG	2. 2 IG v 1 CG	
		3. Sick leave in number of days off in the past 6 weeks	3. 25.5 IG v 22.8 CG	3. 9.6 IG v 9.8 CG	3. 8.8 IG v 6.8 CG	3. 9.8 IG v 5 CG	

*Abbreviations*: *CG* Control group, *CI* Confidence interval, *IG* Intervention group, *OR* odds ratio

**Table 3:** Studies that tested ongoing, intermittently re-enforced implementation interventions

Study	Intervention and control	Outcome measures	Results		Overall intervention success
Becker et al., 2008 [44]	GPE: 3 MFE sessions with feedback for GPs MC: 3 MFE sessions with feedback for GPs and motivational counselling training for practice nurses CG: Postal dissemination of guidelines only	Primary outcome measure:	Primary outcome measure at 6 months:	Primary outcome measure at 12 months:	Partially successful. There was a statistically significant difference between the MC and CG at 6 months on the HFAQ but not at 12 months Days in pain at 6 months in the MC and GPE were also statistically less than the CG, with the GPE maintaining that difference at 12 months Quality of life score was statistically significantly higher in the MC than the CG at 12 months
		Mean score on the HFAQ	72.94 GPE v 73.94 MC v 70.29 CG GPE v CG: CI: −0.704 to 6.007; ρ = 0.12 MC v CG: CI: 0.32 to 6.979; ρ = 0.032*	72.96 GPE v 74.64 MC v 71.56 CG GPE v CG: CI: −2.224 to 5.017; ρ = 0.446 MC v CG: CI: −0.47 to 6.697; ρ = 0.088	
		Secondary outcome measures:	Secondary outcome measures at 6 months:	Secondary outcome measures at 12 months:	
		1. Mean score on the FQPA	1. 36.47 GPE v 36.29 v 33.51 CG GPE v CG: CI: −1.628 to 7.545; ρ = 0.203 MC v CG: CI: −1.784 to 7.347; ρ = 0.23	1. 46.43 GPE v 45.40 MC v 42.88 CG GPE v CG: CI: −1.452 to 8.543; ρ = 0.202 MC v CG: CI: −2.476 to 7.495; ρ = 0.396	
		2. Mean days in pain over the last 6 months.	2. 63.34 GPE v 62.91 MC v 80.78 CG GPE v CG: CI: −26.833 to −6.034; ρ = 0.002* MC v CG: CI: −28.183 to −7.553; ρ = 0.001*	2. 58.48 GPE v 61.57 MC v 71.32 CG GPE v CG: CI: −23.382 to −2.296; ρ = 0.018* MC v CG: CI: −20.198 to −0.689; ρ = 0.067	
		3. Mean days of sick leave in the previous 6 months	3.12.99 GPE v 13.05 MC v 14.34 CG GPE v CG: CI: −5.972 to 3.287; ρ = 0.569 MC v CG: CI: −5.905 to 3.331; ρ = 0.584	3. 6.16 GPE v 6.46 MC v 9.27 CG GPE v CG: CI: −8.582 to 2.358; ρ = 0.256 MC v CG: CI: −8.463 to 2.837; ρ = 0.32	
		4. Quality of life score (Euro Qol)	4. 66.59 GPE v 67.53 MC v 66.85 CG GPE v CG: CI: −2.864 to 2.355; ρ = 0.847 MC v CG: CI: −1.924 to 3.302; ρ = 0.602	4. 68.46 GPE v 70.37 MC v 67.65 CG GPE v CG: CI: −1.736 to 3.344; ρ = 0.535 MC v CG: CI: 0.185 to 5.26; ρ = 0.036*	
		5. FABQ	5. Not expressed as a result	5. Not expressed as a result	
Bishop & Wing, 2006 [28]	IG1: Copy of the guidelines appropriate to management at that time frame sent at assessment; 0–4 weeks; 5–12 weeks and 12 weeks+ IG2: As the IG1 but with patient educational materials at the same stages. CG: No guidelines sent	Primary outcome measure not specified Outcome measures:	Results in percentage of patients:		Largely unsuccessful. The only statistically significant differences between groups were: recommending aerobic exercise in the IG2 v CG and bed rest=/<4 days recommended in the IG1 v CG.
		At assessment:	At assessment:		
		History of initiating event	History of initiating event recorded: 87 % IG1 v 85 % IG2 v 89 % CG *ρ* values not stated		
		Prior history of a similar symptoms	Prior history of similar symptoms recorded: 30 % IG1 v 27 % IG2 v 24 % CG *ρ* values not stated		
		Neurological examination	Neurological examination recorded: 63 % IG1 v 71 % IG2 v 63 % CG *ρ* values not stated		
		Regional back examination	Regional back examination performed recorded: 93 % IG1 v 93 % IG2 v 91 % CG *ρ* values not stated		
		Reference to presence or absence of red flags	Reference to presence or absence of red flags recorded: 4 % IG1 v 4 % IG2 v 5 % GC *ρ* values not stated		
		0-4 weeks:	0-4 weeks:		
		Exercise and reassurance given	Education and reassurance recorded as given: 10 % IG1 v 6 % IG2 v 7 % CG *ρ* values not stated		
		Aerobic exercise promoted	Aerobic exercise recommended recorded: 38 % IG1 v 53 % IG2 v 43 % CG IG2 v CG; ρ = 0.05*		
		Non-narcotic medication prescription	Appropriate medication prescribed: 85 % IG1 v 81 % IG2 v 77 % CG IG1 v CG ρ = 0.14; IG2 v CG ρ = 0.08*		
		Physical therapy modalities usage	Not reported *ρ* values not stated		
		Spinal mobilisation usage	Spinal manipulation usage: 2.5 % IG1 v 5 % IG2 v 6 % CG *ρ* values not stated		
		Bed rest of 4 days or less recommended	Best rest greater than 4 days recommended: 10 % IG1 v 18 % IG2 v17 % GC IG1 v CG ρ = 0.05*; IG2 v CG ρ value not stated		
		5-12 weeks:	5-12 weeks:		
		Work conditioning programmes utilised	Not reported *ρ* values not stated		
		5-12 weeks discordant:	5-12 weeks discordant:		
		Physical therapy modalities usage.	Continued use of PT: 41 % IG1 v 42 % IG2 v 43 % CG *ρ* values not stated Continued use of spinal manipulation: 3 % IG1 v 3 % IG2 v 3 % CG *ρ* values not stated		
		12 weeks + concordant:	12 weeks + concordant:		
		Return to full or modified work.	Not reported ρ values not stated		
		12 weeks + discordant:	12 weeks + discordant:		
		Continued passive therapy or spinal manipulation; recycling through treatments; use of programmes that had previously failed.	Not reported ρ values not stated		
Goldberg et al., 2001 [31]	IG: Surgeon study group meetings; use of local opinion leaders; GP education sessions; printed educational materials; audit; patient educational materials; financial data analysis meetings. CG: Usual care.	Primary outcome measure: Lumbar spine surgery rate per 100,000 adults.	Net reduction in the IG of 20.9 surgeries per 100,000 adults or 8.9 % v CG; ρ = 0.01*		Successful. The difference between the IG and the CG was statistically significant
Kerry et al., 2000 [22]	IG: Guidelines and a covering letter posted at baseline; revised guidelines at 9 months with feedback on referral rates over previous 6 months CG: No guidelines sent	Primary outcome measure: Radiograph request rates.	15 % reduction in the IG compared to 5 % increase in the CG. CI: 3 to 37; ρ ≤ 0.05* but actual value not stated		Successful. The difference between the IG and the CG was statistically significant
Schectman et al., 2003 [23]	E&F: 90-min education session, a copy of the guideline, audit report of patient care during prior year. Follow up at 6 months with a further audit report E&F&PE: As above but with addition of patient educational materials (printed and audio-visual) PE: Patient educational materials only CG: No intervention	Primary outcome measure not specified. Outcome measures:			Unsuccessful. There were no statistically significant differences between groups.
		1. Lumbar spine radiograph request rates	1. 19 % E&F v 18 % CG ρ values not stated		
		2. CT/MRI request rates	2. 5.6 E&F v 7.1 CG ρ values not stated		
		3. Sub-speciality referral rates	3. 8.6 E&F to 7.1 CG ρ values not stated		
		4. PT referral rates	4.10 E&F to 13 in CG No data reported for PE or E&F&PE ρ values not stated		
Winkens et al., 1995 [47]	IG: Regular feedback (x 5 over 2 yrs 7 months) on audit of quantity and quality of diagnostic test referrals CG: No feedback.	Primary outcome measures:			Partially successful. No statistically significant differences between groups in lumbar spine request rate. There was a statistically significant difference between non-rational requests for lumbar spine radiographs pre and post intervention. ρ = 0.004*
		1. Radiograph request rate	1. Not specified but IG before intervention 1128, 1212 at 1 year and 1127 at 2 years. ρ values not stated		
		2. Rate of non-rational requests.	2. % total per Dr: Pre-intervention: 1.92 and 1.38 IG v 1.38 and 1.54 CG 1^st^ data collection point: 0.96 IG v 1.39 CG 2^nd^ DCP: 1.44 IG v 1.22 CG 3^rd^ DCP: IG 0.91 v 1.65 CG 4^th^ DCP: 1.04 IG v 1.24 CG 5^th^ DCP: 1.21 IG v 1.67 CG		

*Abbreviations*: CG control group, *E&F* education and feedback group, *E&F&PE* education and feedback and patient education group, *FABQ* fear avoidance beliefs questionnaire, *FQPA* Freiburg questionnaire physical activity score, *HFAQ* Hannover functional ability questionnaire, *GPE* GP education group, *IG* intervention group, *MC* Motivational counselling group, *MFE* Multifaceted education, *PT* Physical therapy

**Table 4:** Studies testing consistently ongoing implementation interventions

Study	Intervention and control	Outcome measures	Results			Success
Eccles et al., 2001 [24]	IG1: A&F: − audit and feedback IG2: ER - educational reminders IG3: A&F + ER – both interventions. All IGs also received a copy of guidelines CG: Copy of the guidelines only	Primary outcome measure: Radiograph request rate.	−1.53 absolute reduction per 1000 patients ER (95 % CI −2.5 to −0.57) v CG ρ values not stated −0.70 A&F (95 % CI −1.3 to 0.9) v CG ρ values not stated No increased effect A&E + ER.			Successful. Statistically significant differences in lumbar spine radiograph request rate between groups
Ramsey et al., 2003 [48]	IG: Educational reminders CG: Copy of guidelines only	Primary outcome measure: Monthly radiograph request rate for 12 months	Practice mean per month: 1.76 IG v 2.38 CG ρ values not stated 0.64 relative risk (95 % CI 0.43 to 0.96) IG ρ = 0.029*			Successful. Statistically significant differences between groups and no decay of effect over 12 months.
Baker et al., 1987 [50]	IG: Special radiographic requisition form CG: None as a ITS	Primary outcome measure: Radiographic request rate.	Reduction of radiograph request rate from 1443 to 759 ρ values not stated			Successful. A 47 % reduction in the first year maintained for next 3 years
McGuirk et al., 2001 [32]	IG: Special evidence based clinics staffed by motivated practitioners CG: Usual care	Primary outcome measures:	At 3 months:	At 6 months:	At 12 months:	Partially successful. The differences in the VAS at 3 and 12 months were statistically significantly different. The difference between groups in the SF-36 physical functioning at 12 months was statistically ignificantly different. None of these differences in the SF-36 social functioning or physical role were statistically significantly different between groups. The differences in the SF-36 bodily pain at 3 and 6 months were statistically significantly different between groups.
		1. Pain VAS	2 IG v10 CG ρ = 0.001*	3 IG v 4 CG ρ = 0.21	2 IG v 9 CG ρ = 0.042*	
		2. SF-36 physical functioning	1.02 IG v 1.00 CG ρ = 0.364	1.04 IG v 1.04 CG ρ = 0.197	1.07 IG v 0.91 CG ρ = 0.006*	
		2. SF-36 social functioning	1.14 IG v 1.13 CG ρ = 0.853	1.15 IG v 1.13 CG ρ = 0.269	1.15 IG v 1.15 CG ρ = 0.888	
		2. SF-36 physical role	1.17 IG v 1.12 CG ρ = 0.939	1.17 IG v 1.12 CG ρ = 0.35	1.18 IG v 1.12 CG ρ = 0.782	
		2. SF-36 Bodily pain	0.93 IG v 0.79 CG ρ = 0.027*	1.01 IG v 0.90 CG ρ = 0.018*	1.02 IG v 0.90 CG ρ = 0.123	

*Abbreviations*: *A&F* Audit and feedback group, *A&F + ER* audit and feedback and educational reminders group, *CG* control group, *ER* Educational reminders group, *HCP* healthcare practitioners, *IG* intervention group, *ITS* interrupted time series, *VAS* visual analogue scale

### Theoretical models underpinning the implementation intervention

Only one of the 14 included studies described the theoretical underpinning of the implementation intervention chosen. Dey et al. [29] referenced a system of persuasive communication called the Elaboration Likelihood Model of Persuasion [49]. Other studies did use well-known methods of implementation interventions such as audit and feedback [20] or local opinion leaders [30] but the authors did not explicitly provide the rationale for their selection. Given the lack of clarity in most studies about the theoretical models underpinning the selection of implementation interventions, no clear pattern of effectiveness based on the theoretical underpinning of the interventions was observed.

### Number and types of healthcare practitioners studied

A total of 1749 healthcare practitioners were examined in 11 studies giving a mean of 159 practitioners per study with a range of 15 to 462. Four studies did not state the number of healthcare practitioners involved. General Practitioners (GPs)/primary care physicians were the target group of healthcare practitioners in most studies (11 of the 14). Of these, four also included other healthcare practitioners: primary care/practice nurses [44], orthopaedic surgeons and neurosurgeons [31], specialists in rheumatology, rehabilitation and occupational medicine [32], internists and associate practitioners [23]. The other three studies focused on surgical, internal medicine and emergency medicine junior doctors [50] or physiotherapists [26, 30, 45]. There was no clear pattern of effectiveness observed based on the type of healthcare practitioners targeted by the implementation intervention (see Tables 1, 2, 3 and 4).

### Effects of the implementation interventions

Tables 1, 2, 3 and 4 summarise the results of the 14 included studies. The results are presented below, grouped according to the frequency and duration of the implementation interventions since review of the studies highlighted a clear pattern of association between these characteristics and the effectiveness of the implementation intervention.

### Studies that used ‘one-off’ or single implementation interventions

Five studies tested a ‘one-off’ or single event implementation intervention (Table 1) such as one education workshop lasting two hours by Engers et al. [46]. Of these studies, three specified primary outcome measures but none reported a statistically significant difference across all of their measured outcomes between their intervention and comparison. Matowe et al. [25] reported no statistically significant difference; Stevenson et al. [30] reported two statistically significant differences from a total of six outcome measures and Engers et al. [46] reported two statistically significant differences at six weeks out of five patient outcome measures but in the direction favouring the control group and only one at 52 weeks in the direction favouring the intervention group. Dey et al. [29] did not specify a primary outcome measure(s) but reported a statistically significant difference in only one of their five outcome measures; Engers et al. [27] also did not specify a primary outcome measure but reported only one statistically significant difference in the direction favouring the intervention group out of four healthcare practitioner behaviours measured.

### Studies testing short-term implementation interventions with no ongoing implementation effort

Only one study, reported in two papers, used a short-term implementation intervention with no ongoing, implementation effort (Table 2). This comprised an initial two-hour workshop with the postal distribution of printed educational materials and further postal educational materials at 4 and 8 weeks [26, 45]. The first of these papers [26] did not specify a primary outcome measure but reported a moderate change in the intervention group compared to the control across five outcome measures. Four of these were healthcare practitioner behaviours as recorded in the patient record and the fifth measured the number of patient cases for whom all four healthcare practitioner behaviours occurred. However the second paper summarising this study [45] reported no statistically significant differences at 6, 12, 26 or 52 weeks in three primary outcome measures relating to patient outcomes.

### Studies that tested ongoing implementation interventions

The remaining nine studies used ongoing interventions of various types to implement improvements in practice. Of these, six used interventions that intermittently reinforced the implementation effort over time whereas the other three included continual reinforcement of the target behaviour change on a regular sometimes even daily, basis. These two groups of studies are summarised below and in Tables 3 and 4.

### Studies that tested ongoing, intermittently reinforced, implementation interventions

The results reported in these six studies were mixed; at best they reported partial success only across the wide range of outcomes assessed (Table 3). Bishop & Wing [28] studied differences in healthcare practitioner behaviours following their implementation intervention of providing a copy of guidelines appropriate to patient management at different timeframes (at patient assessment, between 0–4 weeks of treatment, between 5–12 weeks and beyond 12 weeks). They reported no statistically significant differences between the intervention and control group in five healthcare practitioner behaviours at the time of patient assessment and only one statistically significant difference after the 0–4 week timeframe out of seven healthcare practitioner behaviours. There were unclear effects after the 5–12 weeks timeframe and no results were presented beyond 12 weeks. Schectman et al. [23] also did not find any statistically significant differences between their two implementation groups versus control in any of the four healthcare practitioner behaviour outcomes they studied. However they stated that the education and feedback group did show a statistically significant increase in overall guideline consistent behaviour compared to control and an overall statistically significant decline of utilisation of services compared to the control. Becker et al. [44] reported moderate success mainly from one type of implementation intervention - motivational counselling training for practice nurses and three education sessions with feedback for GPs. They also tested an intervention that included only the GP education sessions. The difference in the primary outcome measure between the motivational counselling group compared to control (postal dissemination of guidelines only) was statistically significant at 6 months but there were no other statistically significant differences at 6 or 12 months. Both intervention groups showed statistically significant differences in one secondary outcome measure at 6 months versus control but this was only maintained in the intervention group that did not receive motivational counselling at 12 months. Of the other four secondary outcome measures only one demonstrated a statistically significant difference between the motivational counselling group and control at 12 months. Winkens et al. [47] also reported partial success with no reduction in the overall request rates for lumbar spine radiographs but did report a statistically significant reduction in the non-rational lumbar spine radiograph requests in the intervention group compared to control. Two papers reported successful outcomes; Goldberg et al. [31] concluded a reduction in surgery rates in their intervention group compared to control and Kerry et al. [22] reported a large and statistically significant reduction of radiograph requests in their intervention group compared to control. Table 3 summarises the interventions used and their results.

### Studies that tested consistently ongoing implementation interventions

The remaining three studies reporting in four papers utilised at least one component of an implementation intervention that was consistently ongoing over time therefore providing reinforcement of the key behaviour changes desired (Table 4). An example of this type of intervention by Baker et al. [50] was a special request form that had to be completed in order to arrange for the patient to have a lumbar spine radiograph. The key pattern observed across these studies was that they all reported successful outcomes. Eccles et al. [24] reported a statistically significant reduction in lumbar spine radiograph requests compared to control in two of their three implementation groups. Ramsey et al. [48] went on to show that this effect was consistent in the long term over 12 months with no decay of effect. Baker et al. [50] also reported a successful outcome with respect to reduction in lumbosacral radiograph requests, maintained over three years. McGuirk et al. [32] reported a statistically significant reduction in primary outcomes measures of bodily pain at 3 and 6 months and physical function at 12 months as well as a significant reduction in pain at 3 and 12 months. Also a range of secondary outcome measures relating to healthcare professional behaviours and patient outcomes were statistically significantly different between the intervention group and control. Table 4 summarises the interventions and results.

### Risk of bias

Of the 17 papers included in this review, the level of risk of bias was varied; nine were considered to have a low risk of bias, one an unclear risk of bias and seven a high risk of bias.

### Risk of bias in RCTs, non-randomised controlled trials and controlled before-after studies

Of 14 papers, six had a low risk of bias: Bekkering et al. [26]; Dey et al. [29]; Eccles et al. [24]; Engers et al. [46]; Kerry et al. [22]; Winkens et al. [47] – these studies had no or only one category that was classified as at high risk of bias. These high risk categories were considered less important to the integrity of the study – ‘Were incomplete outcome data adequately addressed?’ [26] and [46], ‘Was knowledge of the allocated intervention adequately prevented during study?’ [29]. One study had an unclear risk of bias: Bishop & Wing [28] – this study rated unclear risk in the category of ‘Were baseline outcome measurements similar?’ and high risk in the category of ‘Was the allocation adequately concealed?’ Seven studies had a high risk of bias with all papers rating high risk in two or more categories: Becker et al. [44]; Bekkering et al. [45]; Engers et al. [27]; Goldberg et al. [31]; McGuirk et al. [32]; Schectman et al. [23] and Stevenson et al. [30]. The categories on the risk of bias tool that were rated most frequently as at high risk were: ‘Incomplete outcome data not adequately addressed’ (seven studies), ‘Were baseline outcome measurements similar?’ (five studies) and ‘Were baseline characteristics similar?’ (three studies). Table 5 summarises the results of the risk of bias assessment. Additional file 2 defines how the risk of bias was scored for each category.

**Table 5:** Summary of the risk of bias in RCT, non randomised controlled trials and controlled before-after studies

Risk of bias category	1	2	3	4	5	6	7	8	9	10	11	12	13	14
Was the allocation sequence adequately generated?	U	L	L	L	L	L	L	L	L	U	L	H	U	H
Was the allocation adequately concealed?	L	L	L	L	L	L	U	L	L	U	U	H	U	U
Were baseline outcome measurements similar?	L	U	U	L	U	L	H	L	L	H	H	L	H	H
Were baseline characteristics similar?	L	U	U	L	L	L	L	L	L	H	H	L	H	L
Were incomplete outcome data adequately addressed?	L	L	L	L	H	H	L	H	H	L	U	H	H	H
Was knowledge of the allocated intervention adequately prevented during study?	L	L	L	H	L	U	U	L	L	L	U	U	U	U
Was the study adequately protected against contamination?	L	L	L	L	L	L	L	L	L	L	L	L	L	U
Was the study free from selective outcome reporting?	L	L	L	L	L	L	L	L	L	L	L	L	L	L
Was the study free from other risk of bias?	L	L	L	L	L	L	L	H	H	L	L	L	L	L

*Abbreviations*: 1 – Winkens et al., [47]; 2 – Eccles et al., [24]; 3 – Kerry et al., [22]; 4 – Dey et al., [29]; 5 – Bekkering et al., [26]; 6 – Engers et al., [46]; 7 - Bishop & Wing, [28]; 8 – Bekkering et al., [45]; 9 – Engers et al., [27]; 10 – Stevenson et al., [30]; 11 - Schectman et al., [23]; 12 – McGuirk et al., [32]; 13- Becker et al., [44]; 14 – Goldberg et al., [31]; H – High risk of bias; L – Low risk of bias; U – Unclear risk of bias

### Risk of bias in interrupted time series studies

All of the three papers with this study design had a low risk of bias (Baker et al., [50]; Matowe et al., [25] and Ramsey et al. [48]). Only one category on the risk of bias tool across the three papers was of high risk: ‘Was the intervention independent of other changes?’ (Matowe et al. [25]).

## Discussion

### Summary of results

The key findings of this review show implementation interventions can change healthcare practitioner behaviours to be more in line with best practice recommendations for the management of NSLBP and that some of these interventions are also associated with improvements in patient outcomes. However there was no consistent pattern in terms of the effectiveness of specific types of implementation interventions such as educational events, audit and feedback or use of opinion leaders, of generally passive or active implementation strategies or of targeting specific groups of healthcare practitioners. Rather the results of the 14 included studies showed that implementation intervention effectiveness was more likely to be determined by the frequency and duration of the intervention. Those studies that reported no or very few significant differences in outcome measures all utilised one-off, single or short-term implementation interventions lasting no more than eight weeks. Conversely studies that investigated ongoing and regular implementation interventions demonstrated greater success in changing clinical practice and in sustaining those changes over time. Such successful interventions included those which are traditionally ‘passive’ implementation strategies (such as changing to a new form with which to request radiographs, designed to reduce the number of requests [48]) as well as ‘active’ interventions (such as combined community-wide education and audit processes used to reduce the rates of lumbar spine surgery [31]). The key determinant of success appeared to be the ongoing nature of the intervention rather than the intervention type.

### Implications for clinical practice and research

The evidence from this systematic review suggests that future research focused on improving the translation of best evidence into the clinical management of patients with NSLBP needs to more carefully select and justify the type of implementation intervention(s). One-off, single implementation efforts may be attractive in terms of ease of delivery, low burden for participants, small time commitment and low cost. However this review shows that such one-off, single implementation interventions or even those that last for a short time (up to eight weeks in this review) are unlikely to be successful in changing healthcare practitioner behaviour. Rather the results suggest that ongoing and frequent implementation interventions are required to effectively change clinical practice and improve patient outcomes. This is in line with general recommendations on how to achieve successful implementation [51, 52]. While not always the case, it is likely that such implementation interventions might be costly and time consuming, require changes to the structure of healthcare practitioners’ duties as well as changes to the structure, policies and procedures of healthcare systems. In summary ongoing support may be needed to effect a change in the culture of the individual healthcare practitioners and the organisation within which they work to ensure sustained change in practice that is in line with best available evidence [53]. This would require the updating of practice as current evidence is refined and new evidence comes to light. Such implementation interventions require the co-operation of the target healthcare practitioners, who may not view the implementation as a high priority [54, 55] or may cede to patient requests and by doing so deviate from the best evidence-based recommendations [56].

Ongoing, regular implementation interventions may be costly to organise and sustain yet only two studies of the 14 included in this review reported costs. McGuirk et al. [32] reported that the average cost per patient under evidence-based care was AUS$276 compared to AUS$472 but made no mention of how much their intervention (special evidence based clinics staffed by motivated practitioners) cost to implement. In the study by Goldberg et al. [31] five communities received a complex, multifaceted intervention comprising several information delivery methods over a sustained period of time at an intervention cost of USA$40,000 per community per year. To give some perspective regarding these communities, Goldberg et al. [31] stated that there was an adult population of 123,829 across the five communities, with 7.4 GPs and 4.2 surgeons per 10,000. However, as can be seen from other successful implementation studies such as the introduction and mandatory use of a new radiographic request form by Baker et al. [50], ongoing implementation efforts do not necessarily have to be costly.

Before conducting implementation studies the cost, alterations to daily working practices, changes to workplace policy and procedures and ‘buy in’ or engagement from healthcare practitioners all need to be considered. Given this review highlights the importance of interventions that are ongoing over time, this suggests that a sustainability plan might be needed for after the end of the implementation intervention testing period.

### Behaviour change theory and the evidence-base underpinning the implementation interventions of included studies

The depth and breadth of individual implementation interventions and the different combinations made it challenging to synthesise the data from studies in this review. Although many of the included studies did provide a clear rationale for their selection of implementation interventions it seemed that only certain elements were justified such as the method of knowledge transfer (e.g. via local opinion leaders or by audit and feedback). None of the studies described the basis for other features of their interventions such as frequency and duration, which our review has found to be important.

Behaviour change is required for any implementation intervention to be effective. Michie et al. [57] suggested that better understanding and specifying the behaviour changes required makes implementation interventions more likely to succeed. They also recommended that the elements of the behaviour change intervention should be developed based on the analysis of the antecedents and consequences controlling implementation behaviours and that this analysis should be informed by relevant psychological theory. Examples suggested by Michie and colleagues include the Theoretical Domains Framework, the Capability, Opportunity, Motivation – Behaviour (COM-B) framework and the Behaviour Change Wheel [58]. These processes and behaviour change frameworks were incomplete or absent in the papers included in this review.

Eccles et al. [59] concur with Michie et al. [57] and provide examples of three types of psychological theory: motivational (how individuals come to wish/intend to change behaviour), action (how individuals move from intention to actual behaviour) and stage theory, which proposes an orderly progression through discrete stages towards behaviour change. Stage theory in particular appears to underpin the most important features of successful implementation interventions.

### Risk of bias of included papers

The risk of bias results for the 17 included papers does weaken the conclusions from this review. In total 7 or 41 % of papers were rated as having a high risk of bias. These include the papers by Becker et al. [44], Goldberg et al. [31], McGuirk et al. [32] and Schectman et al. [23] all of which reported at least moderately successful outcomes. Of these four papers, three were rated at high risk of bias in the category, ‘were incomplete outcome data adequately addressed?’ and the other paper (Schectman et al., [23] was rated as unclear for this category. Rating high risk in this category clearly affects the confidence in the results. These four papers also scored unclear risk for the category ‘was knowledge of the allocated intervention adequately prevented during the study?’ meaning that they did not specify this information in their paper.

### Strengths and limitations

The methods of this review were developed in conjunction with experts in the field and followed the guidance from the Cochrane Collaboration and EPOC. The electronic database searching was thorough and followed that suggested by the Cochrane Handbook for Systematic Reviews of Interventions [60]. Study eligibility and risk of bias of included studies were determined through independent assessment by members of the study team and in line with Cochrane guidance [59]. The main limitation of this review is the variable risk of bias within the included published papers. Adhering to the EPOC guidance for study eligibility meant that many studies were excluded based on their study design. In addition the included studies were heterogeneous in terms of study design, type of implementation intervention used, healthcare practitioners targeted, practitioner behaviour targeted and study setting. This heterogeneity made it challenging to synthesise the results. Clearly the quality of research in this field varies and further high quality studies of implementation interventions in the field of NSLBP are needed. One further limitation of note is that only papers published in English were considered for inclusion.

## Conclusion

The results of this review indicate that the most successful interventions to support implementation of best available evidence into clinical practice for NSLBP are those that occur more frequently and are ongoing. Other factors such as intervention type, complexity or target healthcare practitioner or behaviour did not appear to determine the success of the implementation intervention tested. These results must be interpreted with some caution given that many included papers were at high risk of bias. Further high quality studies are needed to robustly test the effectiveness of implementation interventions in this field. The investigators of future implementation studies in this area should develop a strong rationale for the implementation intervention(s) chosen by identifying barriers and facilitators to implementation of best available evidence, select relevant implementation interventions to overcome these barriers and enhance the facilitators and follow best practice guidelines in design, conduct and reporting of their studies. In particular future studies need to give careful consideration to the frequency and duration of their implementation intervention and evaluate cost-effectiveness.

## Abbreviations

ASSIA, Applied Social Science Index and Abstracts; EPOC, The Cochrane Effective Practice and Organisation of Care Group; LBP, Low back pain; NSLBP, Non-specific low back pain; PICO, Population; Intervention; Comparator and Outcome; PRISMA, Preferred Reporting Items for Systematic Reviews and Meta-analyses; RCT, Randomised controlled trial; SF-36, Short Form 36; VAS, Visual analogue scale; WHO, World Health Organisation

## Additional files

Additional file 1: MEDLINE search. (DOCX 82 kb) Additional file 2: Suggested risk of bias criteria for EPOC reviews. (DOCX 96 kb) Additional file 3: Reasons for exclusion of the 136 full text papers. (DOCX 142 kb)
