# Supplementary material for: Implementation interventions to improve the management of non-specific low back pain: a systematic review
Source: BMC Musculoskelet Disord. 2016 Jun 10;17:258. doi: 10.1186/s12891-016-1110-z (PMC4902903; doi:10.1186/s12891-016-1110-z)
Supplement: Additional file 1: — MEDLINE search. (DOCX 82 kb) [file 12891_2016_1110_MOESM1_ESM.docx]

**MEDLINE search:**

Limits: Date range 1987 to December 31^st^ 2012; English language, adult, human.

Search terms in ‘Any field’:

1: “back pain” – mapped to thesaurus and exploded with back pain.

2: “low* back pain” – mapped to thesaurus and exploded with low back pain and back pain.

3: ”lumbar spine pain” – mapped to thesaurus and exploded with low back pain.

4: ”non-specific low* back pain” – mapped to thesaurus and exploded with low back pain and back pain.

5: ”back ache” – mapped to thesaurus and exploded with back pain.

6: ”low*back ache” – mapped to thesaurus and exploded with low back pain.

7: “simple low* back pain” – mapped to thesaurus and exploded with low back pain and back pain.

8: Searches 1-7 combined with OR.

9: “healthcare quality assurance” – mapped to thesaurus and exploded with quality assurance, health care.

10: ”healthcare quality assessment”

11: “clinical practice guidelines”

12: ”clinical guidelines”

13: “best practice evidence” – mapped to thesaurus and exploded with evidence based medicine.

14: ”translation of best evidence”

15: ”research evidence” – mapped to thesaurus and exploded with evidence based medicine

16: “quality improvement” – mapped to thesaurus and exploded with quality improvement.

17: ”effectiveness study”

18: “practice feedback” – mapped to thesaurus and exploded with feedback.

19: “evidence-based education* program*”

20: ”evidence-based management” – mapped to thesaurus and exploded with evidence-based practice.

21: ”evidence-based practice” – mapped to thesaurus and exploded with evidence-based practice.

22: “clinical audit” – mapped to thesaurus and exploded with clinical audit and medical audit.

23: “multi-faceted implement* program*”

24: ”mass media implement* program*” – mapped to thesaurus and exploded with mass media and health promotion.

25: ”implement* program*”

26: ”theory based implement*”

27: “theory based intervention”

28: ”implement* intervention”

29: intervention

30: “patient outcomes”

31: “implement* effort”

32: ”implement* strategy”

33: ”guideline implement*” – mapped to thesaurus and exploded with guideline adherence.

34: “effective implement*”

35: implement*

36: ”best practice implement*”

37: ”evidence into practice” – mapped to thesaurus and exploded with evidence based medicine.

38: “healthcare professional behavio* change”

39: ”behavio* change”

40: “translational research” – mapped to thesaurus and exploded with translational medical research.

41: ”research into practice”

42: ”knowledge translation”

43: ”adoption of best practice”

44: 9-43 combined with OR.

45: 8 and 44 combined with AND.
