# Supplementary material for: Implementation interventions to improve the management of non-specific low back pain: a systematic review
Source: BMC Musculoskelet Disord. 2016 Jun 10;17:258. doi: 10.1186/s12891-016-1110-z (PMC4902903; doi:10.1186/s12891-016-1110-z)
Supplement: Additional file 3: — Reasons for exclusion of the 136 full text papers. (DOCX 142 kb) [file 12891_2016_1110_MOESM3_ESM.docx]

1. Ahles TA., Wasson JH., Seville JL., Johnson DJ., Cole BF., Hanscom B., Stukel TA., McKinstry E. (2006) A controlled trial of methods for managing pain in primary care patients with or without co-occurring psychosocial problems. Annals of Family Medicine 4(4):341-350.

Excluded as did not assess healthcare practitioner behaviour change/healthcare system change or effect on patient outcomes.

2. Ammendolia C., Cassidy D., Steensta I., Soklaridis S., Boyle E., Eng S., Howard H., Bhupinder B., Cote P. (2009) Designing a workplace return-to-work program for occupational low back pain: an intervention mapping approach. BMC Musculoskeletal Disorders 10:65-65.

Excluded as no actual implementation effort performed.

3. Armstrong MP., McDonough S., Baxter GD. (2003) Clinical guidelines versus clinical practice in the management of low back pain. International Journal of Clinical Practice 57(1):9-13.

Excluded as no actual implementation effort performed.

4. Barnett AG., Underwood MR., Vickers MR. (1999) Effect of UK national guidelines on services to treat patients with acute low back pain: follow up questionnaire survey. British Medical Journal 318(7188):919-20.

Excluded as no actual implementation effort performed.

5. Barton C., Breslin A., Fleur J., Jones J., Lewis R., McSweeney L., Maddison P., Norgain C., Smith S., Thomas C., Tillson C. (2004) Improved access and targeting of musculoskeletal services in northwest Wales: Targeted Early Access to Musculoskeletal Services (TEAMS) programme: quality improvement report. British Medical Journal 329(7478):1325-27.

Excluded as no actual implementation effort performed.

6. Becker A., Held H., Redaelli M. (2012) Implementation of a guideline for low back pain management in primary care: a cost-effectiveness analysis. Spine 37(8):701-710.

Excluded as did not assess of healthcare practitioner behaviour change/healthcare system change or effect on patient outcomes.

7. Becker A., Leonhardt C., Kochen MM., Keller S., Wegscheider K., Baum E., Donner-Banzhoff N., Pfingsten M., Hildebrandt J., Basler HD., Chenot JF. (2008) Effects of two guideline implementation strategies on patient outcomes in primary care: a cluster randomized controlled trial. Spine 33(5):473-80.

Duplicate of the included study: Becker A., Leonhardt C., Kochen MM. (2008) Effects of two guideline implementation strategies on patient outcomes in primary care: a cluster randomized controlled trial. Spine 33(5):473-480.

8. Bishop P., Quon J., Olson D., Nixdorf D., Yee B., Fisher C., Dvorak M. (2007) A randomized controlled trial on the effectiveness of clinical practice guidelines in the medical and chiropractic management of patients with acute mechanical lower back pain. Canadian Journal of Surgery 50(June Suppl.):S4-S13.

Excluded as abstract only.

9. Bishop P., Fisher C., Quon J., Dvorak M. (2008) Clinical practice guideline-based treatment is not effective for all patients with acute lower back pain. Canadian Journal of Surgery 51(Suppl) S1-23

Excluded as abstract only.

10. Bishop PB., Quon JA., Fisher CG. & Dvorak MF. (2010) The Chiropractic Hospital-based Interventions Research Outcomes (CHIRO) study: a randomized controlled trial on the effectiveness of clinical practice guidelines in the medical and chiropractic management of patients with acute mechanical low back pain. Spine Journal 10(12):1055-64.

Excluded as no actual implementation effort made.

11. Breen AC., Carr E., Langworthy JE., Osmond C. & Worswick L. (2011) Back pain outcomes in primary care following a practice improvement intervention:- a prospective cohort study. BMC Musculoskeletal Disorders 12:28.

Excluded as an uncontrolled before and after study.

12. Buchbinder R,. & Jolley D. (2004) Population based intervention to change back pain beliefs: three year follow up population survey. British Medical Journal 328(7435).

Excluded as a controlled before and after study but with only one control site.

13. Buchbinder R. & Jolley D. (2005) Effects of a media campaign on back beliefs is sustained 3 years after its cessation. Spine 30(11):1323-1330.

Excluded as a controlled before and after study but with only one control site.

14. Buchbinder R. & Jolley D. (2007) Improvements in general practitioner beliefs and stated management of back pain persist 4.5 years after the cessation of a public health media campaign. Spine 32(5):E156-E162.

Excluded as a controlled before and after study but with only one control site.

15. Buchbinder R. (2008) Self-management education en masse: effectiveness of the Back Pain: Don’t take it lying down mass media campaign. Medical Journal of Australia 189:S29-S32

Excluded as only a commentary paper.

16. Buchbinder R., Jolley D. & Wyatt M. (2001) Volvo Award winner in clinical studies: effects of a media campaign on back pain beliefs and its potential influence on management of low back pain in general practice. Spine 26(23):2535-2542.

Excluded as a controlled before and after study but with only one control site.

17. Buhrman M., Faltenhag S., Strom L. & Andersson G. (2004) Controlled trial of Internet-based treatment with telephone support for chronic back pain. Pain 111(3):368-77.

Excluded as no actual implementation effort performed.

18. Butler RJ., Johnson WG. & Gray BP. (2007) Timing makes a difference: early nurse case management intervention and low back pain. Professional Case Management 12(6):316-327; quiz 28-9.

Excluded as an un-controlled study.

19. Chenot J-F., Scherer M., Becker A., Donner-Banzhoff N., Baum E., Leonhardt C., Keller S., Pfingsten M., Hildebrandt J., Basler H-D. & Kochen MM. (2008) Acceptance and perceived barriers of implementing a guideline for managing low back in general practice. Implementation Science 3:7.

Excluded as a survey only study.

20. Cherkin D., Deyo RA., Berg AO., Bergman JJ. & Lishner DM. (1991) Evaluation of a physician education intervention to improve primary care for low-back pain I. Impact on physicians. Spine 16(10):1168-72.

Excluded as an uncontrolled before and after study.

21. Corson K., Doak MN., Denneson L., Crutchfield M., Soleck G., Dickinson KC., Gerrity MS. & Dobscha SK. (2011) Primary Care Clinician Adherence to Guidelines for the Management of Chronic Musculoskeletal Pain: Results from the Study of the Effectiveness of a Collaborative Approach to Pain. Pain Medicine 12(10):1490-501.

Excluded as not back pain specific, mixes musculoskeletal conditions.

22. Cretin S., O’Farley D., Dolter KJ. & Nicholas W. (2001) Evaluating an integrated approach to clinical quality improvement. Medical Care 39(8)Suppl. 2:II-70-II-84

Excluded as an uncontrolled before and after study.

23. Cunningham CG., Flynn TA., Toole CM., Ryan RG., Gueret PWJ., Bulfin S., Seale O. & Blake C. (2008) Working Backs Project-implementing low back pain guidelines. Occupational Medicine 58(8):580-83.

Excluded as an uncontrolled before and after study.

24. Curtis P., Carey TS., Evans P., Rowane MP., Jackman A. & Garrett J. (2000) Training in back care to improve outcome and patient satisfaction. Teaching old docs new tricks. Journal of Family Practice 49(9):786-92.

Excluded as an observational cohort study.

25. Davies S., Quinter J., Parsons R., Parkitny L., Knight P., Forrester E., Roberts M., Graham C., Visser E., Antill T., Packer T. & Schug SA. (2011) Pre-clinic group education sessions reduce waiting times and costs at public pain medicine units. Pain Medicine 12:59-71

Excluded as not specific to non-specific low back pain.

26. Derebery VJ., Giang GM., Saracino G. & Fogarty WT. (2002) Evaluation of the impact of a low back pain educational intervention on physicians' practice patterns and patients' outcomes. Journal of Occupational & Environmental Medicine 44(10):977-84.

Excluded as a controlled before and after study but with less than two intervention and two control sites.

27. De Vos Meiring P. & Wells IP. (1990) The effect of radiology guidelines for General Practitioners in Plymouth. Clinical Radiology 42:327-329

Excluded as a time-interrupted series but with only two measurements pre-intervention and one post intervention.

28. Deyo RA. (1993) Clinical strategies for controlling costs and improving quality in the primary care of low back pain. Journal of Back and Musculoskeletal Rehabilitation 3(4):1-13.

Excluded as a review paper.

29. Deyo RA., Schall M., Berwick DM., Nolan T. & Carver P. (2000) Continuous quality improvement for patients with back pain. Journal of General Internal Medicine 15(9):647-55.

Excluded as an uncontrolled before and after study.

30. Dobscha SK., Corson K., Leibowitz RQ., Sullivan MD. & Gerrity MS. (2008) Rationale, design, and baseline findings from a randomized trial of collaborative care for chronic musculoskeletal pain in primary care. Pain Medicine 9(8):1050-64.

Excluded as an earlier version of 31.

31. Dobscha SK., Corson K., Perrin NA., Hanson GC., Leibowitz RQ., Doak MN., Dickinson KC., Sullivan MD. & Gerrity MS. (2009) Collaborative Care for Chronic Pain in Primary Care A Cluster Randomized Trial. Journal of the American Medical Association 301(12):1242-52.

Excluded as back pain results are not presented separately.

32. Donovan MI., Evers K., Jacobs P. & Mandleblatt S. (1999) When there is no benchmark: Designing a primary care-based chronic pain management program from the scientific basis up. Journal of pain and symptom management 18(1):38-48.

Excluded as no actual implementation effort made.

33. Douglas TS., Mann NH. & Hodge AL. (1998) Evaluation of preoperative patient education and computer-assisted patient instruction. Journal of Spinal Disorders 11(1):29-35.

Excluded as not non-specific low back pain patients – patients who were listed for discectomy.

34. Driessen M., Bosmans J., Proper K., Anema J., Bongers P. & van der Beek A. (2012) The economic evaluation of a Participatory Ergonomics programme to prevent low back and neck pain. Work-a Journal of Prevention Assessment & Rehabilitation 41:2315-20.

Excluded as no actual implementation made.

35. Driessen MT., Proper KI., Anema JR., Knol DL., Bongers PM. & van AJB. (2011) The effectiveness of participatory ergonomics to prevent low-back and neck pain - results of a cluster randomized controlled trial. Scandinavian Journal of Work, Environment and Health 37(5):383-93.

Excluded as no actual implementation effort made.

36. Ersek M., Turner JA., Cain KC. & Kemp CA. (2008) Results of a randomized controlled trial to examine the efficacy of a chronic pain self-management group for older adults. Pain 138(1):29-40.

Excluded as no actual implementation effort to made.

37. Ersek M., Turner JA., McCurry SM., Gibbons L. & Kraybill BM. (2003) Efficacy of a self-management group intervention for elderly persons with chronic pain. Clinical Journal of Pain 19(3):156-67.

Excluded as 36.

38. Escolar-Reina P., Medina-Mirapeix F., Gascon-Canovas JJ., Montilla-Herrador J., Valera-Garrido JF. & Collins SM. (2009) Self-management of chronic neck and low back pain and relevance of information provided during clinical encounters: an observational study. Archives of Physical Medicine & Rehabilitation 90(10):1734-9.

Excluded as no actual implementation effort made.

39. Evans DW., Breen AC., Pincus T., Sim J., Underwood M., Vogel S. & Foster NE. (2010) The effectiveness of a posted information package on the beliefs and behavior of musculoskeletal practitioners: The UK chiropractors, osteopaths, and musculoskeletal physiotherapists low back pain managemENT (COMPLeMENT) randomized trial. Spine 35(8):858-66.

Excluded as actual healthcare practitioner behaviour change not assessed only changes to intended behaviours and beliefs.

40. Faber E., Bierma-Zeinstra SM., Burdorf A., Nauta AP., Hulshof CT., Overzier PM., Miedema HS. & Koes BW. (2005) In a controlled trial training general practitioners and occupational physicians to collaborate did not influence sick leave of patients with low back pain. Journal of clinical epidemiology 58(1):75-82.

Excluded as although a controlled cluster trial, only one intervention and one study site.

41. Ferguson F., Holdsworth L. & Rafferty D. (2010) A national framework for supporting improvements in the physiotherapy assessment and management of low back pain: the Scottish experience. Physiotherapy 96(3):198-205.

Excluded as an uncontrolled before and after study.

42. Feuerstein M., Hartzell M., Rogers HL. & Marcus SC. (2006) Evidence-based practice for acute low back pain in primary care: patient outcomes and cost of care. Pain 124(1-2):140-9.

Excluded as a time-interrupted series but with only one pre-intervention measure.

43. Ferreira MS. & Navega MT. (2010) Effects of a Guidance Program to Adults with Low Back Pain. Acta Ortopedica Brasileira 18(3):127-31.

Excluded as not an implementation effort directed at changing healthcare practitioner behaviour.

44. Flavell HA., Carrafa GP., Thomas CH. & Disler PB. (1996) Managing chronic back pain: Impact of an interdisciplinary team approach. Medical Journal of Australia 165(5):253-55.

Excluded as no implementation effort made to change healthcare practitioner behaviour.

45. Fleten N. & Johnsen R. (2006) Reducing sick leave by minimal postal intervention: a randomised, controlled intervention study. Occupational & Environmental Medicine 63(10):676-82.

Excluded as no implementation effort made to change healthcare practitioner behaviour.

46. Fleuren M., Dusseldorp E., van den Bergh S., Vlek H., Wildschut J., van den Akker E. & Wijkel D. (2010) Implementation of a shared care guideline for back pain: effect on unnecessary referrals. International Journal for Quality in Health Care 22(5):415-20.

Excluded as this looked at a specific type of low back pain (that with radicular pain) not non-specific low back pain.

47. Foster NE., Mullis R., Young J., Doyle C., Lewis M., Whitehurst D. & Hay EM. (2010) IMPaCT Back study protocol. Implementation of sub-grouping for targeted treatment systems for low back pain patients in primary care: a prospective population-based sequential comparison. BMC Musculoskeletal Disorders 11:186

Excluded as study is incomplete.

48. Franklin GM., Mai J., Turner J., Sullivan M., Wickizer T. & Fulton-Kehoe D. (2012) Bending the prescription opioid dosing and mortality curves: Impact of the Washington State opioid dosing guideline. American Journal of Industrial Medicine 55(4):325-31.

Excluded as not related to non-specific low back pain.

49. Freeborn DK., Shye D., Mullooly JP., Eraker S. & Romeo J. (1997) Primary care physicians’ use of lumbar spine imaging tests. Journal of General and Internal Medicine 12:619-625

Excluded as although a controlled before and after study but with only one intervention and control site.

50. Fritz JM., Cleland JA. & Brennan GP. (2007) Does adherence to the guideline recommendation for active treatments improve the quality of care for patients with acute low back pain delivered by physical therapists? Medical Care 45(10):973-80.

Excluded as no implementation effort made to change healthcare practitioner behaviour.

51. Fruth SJ., Van Veld RD., Despos CA., Martin RD., Hecker A. & Sincroft EE. (2010) The influence of a topic-specific, research-based presentation on physical therapists' beliefs and practices regarding evidence-based practice. Physiotherapy Theory & Practice 26(8):537-57.

Excluded as not specifically to non-specific low back pain.

52. Gilbey A. (2011) Evidence-based clinical practice guidelines vs. family physician usual care for the treatment of acute lower back pain: What is the role of chiropractic? Focus on Alternative and Complementary Therapies 16(3):243-44.

Excluded as only commentary another study.

53. Gonzalez-Urzelai V., Palacio-Elua L. & Lopez-de-Munain J. (2003) Routine primary care management of acute low back pain: adherence to clinical guidelines. European Spine Journal 12(6):589-94.

Excluded as no actual implementation effort made.

54. Gross DP. & Lowe A. (2009) Evaluation of a knowledge translation initiative for physical therapists treating patients with work disability. Disability and rehabilitation 31(11):871-79.

Excluded as not specific to non-specific low back pain.

55. Gross DP., Russell AS., Ferrari R., Battie MC., Schopflocher D., Hu R., Waddell G. & Buchbinder R. (2010) Evaluation of a Canadian back pain mass media campaign. Spine 35(8):906-13.

Excluded as although a controlled before and after study there was only one control site.

56. Gutierrez B. & Kurlantzick V. (1997) Using low back pain guidelines to change health care professional practice patterns. Association for Health Services Research 1997;14:105-106

Excluded as abstract only.

57. Haas M., Groupp E., Muench J., Kraemer D., Brummel-Smith K., Sharma R., Ganger B., Attwood M. & Fairweather A. (2005) Chronic disease self-management program for low back pain in the elderly. Journal of Manipulative & Physiological Therapeutics 28(4):228-37.

Excluded as did not assess healthcare practitioner change, health system change or effect on patient outcomes.

58. Hay EM., Mullis R., Lewis M., Vohora K., Main CJ., Watson P., Dziedzic KS., Sim J., Lowe CM. & Croft PR. (2005) Comparison of physical treatments versus a brief pain-management programme for back pain in primary care: a randomised clinical trial in physiotherapy practice. Lancet 365(9476):2024-30.

Excluded as did not assess healthcare practitioner change, health system change or effect on patient outcomes.

59. Hazard RG., Reid S., Haugh LD. & McFarlane G. (2000) A controlled trial of an educational pamphlet to prevent disability after occupational low back injury. Spine 25(11):1419-23.

Excluded as did not assess healthcare practitioner change, health system change or effect on patient outcomes.

60. Hendriks EJM., Kerssens JJ., Dekker J., Nelson RM. & Oostendorp RAB. (2003) One-time physical therapist consultation in primary health care. Physical Therapy 83(10):918-31.

Excluded as did not assess healthcare practitioner behaviour change, healthcare system change or effect on patient outcomes.

61. Hill JC, Whitehurst DG, Lewis M, Bryan S, Dunn KM, Foster NE, Konstantinou K, Main CJ, Mason E, Somerville S, Sowden G, Vohora K, Hay EM. Comparison of stratified primary care management for low back pain with current best practice (STarT Back): a randomised controlled trial. Lancet 2011;378(9802):1560-71.

Excluded as not specific to non-specific low back pain (mixed back pain with and without radiculopathy).

62. Hoeijenbos M., Bekkering T., Lamers L., Hendriks E. & van M, Koopmanschap M. (2005) Cost-effectiveness of an active implementation strategy for the Dutch physiotherapy guideline for low back pain. Health Policy 75(1):85-98.

Excluded as did not assess healthcare practitioner change, health system change or effect on patient outcomes.

63. Hollingworth W., Todd CJ., King H., Males T., Dixon AK., Karia KR. & Kinmonth AL. (2002) Primary care referrals for lumbar spine radiography: diagnostic yield and clinical guidelines. British Journal of General Practice 52(479):475-80.

Excluded as the study didn’t attempt an implementation rather assessed before and after guidelines were disseminated by governing body.

64. Ihlebaek C. & Eriksen HR. (2005) Myths and perceptions of back pain in the Norwegian population, before and after the introduction of guidelines for acute back pain. Scandinavian Journal of Public Health 33(5):401-6.

Excluded as did not assess healthcare practitioner change, health system change or effect on patient outcomes.

65. Ijzelenberg H., Meerding WJ. & Burdorf A. (2007) Effectiveness of a back pain prevention program: a cluster randomized controlled trial in an occupational setting. Spine 32(7):711-9.

Excluded as did not assess healthcare practitioner change, health system change or effect on patient outcomes.

66. Iles R. (2011) A randomized controlled trial of telephone coaching for return to usual activity in low back pain. Spine Journal 11(10).

Excluded as did not assess healthcare practitioner change, health system change or effect on patient outcomes.

67. Iles R., Taylor NF., Davidson M. & O'Halloran P. (2011) Telephone coaching can increase activity levels for people with non-chronic low back pain: a randomised trial. Journal of Physiotherapy 57(4):231-8.

As 66.

68. Jackson JL. & Browning R. (2005) Impact of national low back pain guidelines on clinical practice. Southern medical journal 98(2):139-43.

Excluded as no actual implementation effort made but assessment made before and after national guidelines were released.

69. Jeannot J-G., Scherer F., Pittet V., Burnand B. & Vader J-P. (2003) Use of the World Wide Web to Implement Clinical Practice Guidelines: A Feasibility Study. Journal of Medical Internet Research 5(2):e12-e12.

Excluded as not non-specific low back pain (indications for back surgery).

70. Jellema P. & Daw He. (2005) Should treatment of (sub)acute low back pain be aimed at psychosocial prognostic factors? Cluster randomised clinical trial in general practice. British Medical Journal 331(7508):84-87

Excluded as no assessment of healthcare practitioner behaviour change, healthcare system change or effect on patient outcomes.

71. Keijsers JF., Bouter LM., Meertens RM., Kessels AG. & Knipschild PG. (1992) The impact of back school research on the beliefs of health care professionals: a randomised survey of general practitioners and physiotherapists. Physiotherapy Theory and Practice 8(2):79-83.

Excluded as no assessment of healthcare practitioner behaviour change, healthcare system change or effect on patient outcomes. Assessed changes to beliefs only.

72. Keijsers JF., Steenbakkers MW., Meertens RM., Bouter LM. & Kok G. (1990) The efficacy of the back school: a randomised trial. Arthritis Care and Research 3(4):204-9.

Excluded as a study on the efficacy of back schools and not an implementation study.

73. Kendrick D., Fielding K., Bentley E., Kerslake R., Miller P. & Pringle M. (2001) Radiography of the lumbar spine in primary care patients with low back pain: randomised controlled trial. British Medical Journal 322(7283):400-5.

Excluded as no actual implementation effort made.

74. Klein BJ., Radecki RT., Foris MP., Fell EI. & Hickey ME. (2000) Bridging the gap between science and practice in managing low back pain - A comprehensive spine care system in a health maintenance organization setting. Spine 25(6):738-40.

Excluded as an uncontrolled before and after study.

75. Kovacs F., Abraira V., Santos S., Diaz E., Gestoso M., Muriel A., Gil MTM., Mufraggi N., Noguera J., Zamora J. & Spanish BN. (2007) A comparison of two short education programs for improving low back pain-related disability in the elderly: a cluster randomized controlled trial. Spine32(10):1053-9.

Excluded as no assessment of healthcare practitioner behaviour change, healthcare system change or effect on patient outcomes.

76. Lambeek LC., Anema JR., van Royen JB., Buijs PC., Wuisman PI, van Tulder MW., van MechelenW. (2007) Multidisciplinary outpatient care program for patients with chronic low back pain: design of a randomized controlled trial and cost-effectiveness study. BMC Public Health 7:254.

Excluded as a comparison of two interventions rather than an implementation study.

77. Levsen MJ., Hansen ML., Kent AD., Sieren JJ., Thoreson JP. & Farrell KP. (2001) Effects of physical therapist training on outcomes of patients with chronic low back pain or chronic shoulder pain. Journal of Manual and Manipulative Therapy 9(2):84-91

Excluded as not an implementation of best research evidence.

78. Loisel P., Gosselin L., Durand P., Lemaire J., Poitras S. & Abenhaim L. (2001) Implementation of a participatory ergonomics program in the rehabilitation of workers suffering from sub-acute back pain. Applied Ergonomics 32(1):53-60.

Excluded as no assessment of healthcare practitioner behaviour change, healthcare system change or effect on patient outcomes.

79. Loisel P., Hong QN., Imbeau D., Lippel K., Guzman J., MacEachen E., Corbiere M., Santos BR., Anema JR (2009). The Work Disability Prevention CIHR Strategic Training Program: Program Performance After 5 Years of Implementation. Journal of Occupational Rehabilitation 19(1):1-7.

Excluded as no assessment of healthcare practitioner behaviour change, healthcare system change or effect on patient outcomes.

80. Loisel P., Lemaire J., Poitras S., Durand MJ., Champagne F., Stock S., Diallo B. & Tremblay C. (2002) Cost-benefit and cost-effectiveness analysis of a disability prevention model for back pain management: a six year follow up study. Occupational & Environmental Medicine 59(12):807-15.

Excluded as no assessment of healthcare practitioner behaviour change, healthcare system change or effect on patient outcomes.

81. Lugtenberg M., Burgers JS. & Westert GP. (2009) Effects of evidence-based clinical practice guidelines on quality of care: a systematic review. Quality & Safety in Health Care 2009;18(5):385-92.

Excluded as no actual implementation effort made.

82. McCluskey S., Burton AK. & Main CJ. (2006) The implementation of occupational health guidelines principles for reducing sickness absence due to musculoskeletal disorders. Occupational Medicine 56(4):237-42.

Excluded as no assessment of healthcare practitioner behaviour change, healthcare system change or effect on patient outcomes.

83. McCracken LM., Boichat C. & Eccleston C. (2012) Training for General Practitioners in opioid prescribing for chronic pain based on practice guidelines: A randomized pilot and feasibility trial. Journal of Pain 13(1):32-40.

Excluded as not assessing non-specific low back pain.

84. McKenzie JE., French SD., O'Connor DA., Grimshaw JM., Mortimer D., Michie S., Francis J., Spike N., Schattner P., Kent PM., Buchbinder R. & Green SE. (2008) IMPLEmenting a clinical practice guideline for acute low back pain evidence-based manageMENT in general practice (IMPLEMENT): Cluster randomised controlled trial study protocol. Implementation Science 3(11).

Excluded as study incomplete.

85. McKenzie JE., O'Connor DA., Page MJ., Mortimer DS., French SD., Walker BF., Keating JL., Grimshaw JM., Michie S., Francis JJ. & Green SE. (2010) Improving the care for people with acute low-back pain by allied health professionals (the ALIGN trial): A cluster randomised trial protocol. Implementation Science 5:86.

Excluded as study incomplete.

86. Mortimer D., French SD., McKenzie JE., O'Connor DA. & Green SE. (2008) Implement Study G. Protocol for economic evaluation alongside the IMPLEMENT cluster randomised controlled trial. Implementation Science 3:12.

Excluded as study incomplete.

87. Mikulich VJ., Liu YCA., Steinfeldt J. & Schriger DL. (2001) Implementation of clinical guidelines through an electronic medical record: physician usage, satisfaction and assessment. International Journal of Medical Informatics 63(3):169-78.

Excluded as an uncontrolled before and after study.

88. Miller J., Pinnington MA. & Stanley I. (2004) An evaluation of prompt access to physiotherapy in the management of low back pain in primary care. Family Practice 21(4):372-80.

Excluded as an uncontrolled before and after study.

89. Moessner M., Schiltenwolf M. & Neubauer E. (2012) Internet-based aftercare for patients with back pain - A pilot study. Telemedicine and E-Health 18(6):413-19.

Excluded as no assessment of healthcare practitioner behaviour change, healthcare system change or effect on patient outcomes.

90. Morasco BJ., Duckart JP. & Dobscha SK. (2011) Adherence to clinical guidelines for opioid therapy for chronic pain in patients with substance use disorder. Journal of General Internal Medicine 26(9):965-71.

Excluded as a survey study.

91. Mo-Yee P., Hung-Kay D. & Pope MH. (2008) Early physiotherapy intervention in an Accident and Emergency Department reduces pain and improves satisfaction for patients with acute low back pain: a randomised trial. Australian Journal of Physiotherapy 54(4):243-249.

Excluded as no actual implementation effort made.

92. Negrini S., Monticone M., Chirchiglia S., Fabiani L., Gattinoni F., Giorgianni R., Giovannoni S., Minozzi S. & Politano E. (2004) Experience in Italy in the development and application of clinical guidelines for low-back pain. Europa Medicophysica 40(1):45-53.

Excluded as not an actual implementation study but a review of other studies.

93. Negrini S., Politano E., Carabalona R. & Mambrini A. (2001) General practitioners' management of low back pain - Impact of clinical guidelines in a non-English-speaking country. Spine 26(24):2727-33.

Excluded as a cross sectional study.

94. Nordeman L., Nilsson B., Moller M. & Gunnarsson R. (2006) Early access to physical therapy treatment for sub-acute low back pain in primary health care: a prospective randomized clinical trial. Clinical Journal of Pain 22(6):505-11.

Excluded as no assessment of healthcare practitioner behaviour change, healthcare system change or effect on patient outcomes.

95. Oakeshott P., Kerry SM. & Williams JE. (1994) Randomized controlled trial of the effect of the Royal College of Radiologists’ guidelines on general practitioners’ referrals for radiographic examination. British Journal of General Practitioners 44(382):197–200.

Excluded as mixed spinal results, not low back specific. Lead author contacted but no separate results available.

96. Overmeer T., Linton SJ., Holmquist L., Eriksson M. & Engfeldt P. (2005) Do evidence-based guidelines have an impact in primary care? A cross-sectional study of Swedish physicians and physiotherapists. Spine 30(1):146-51.

Excluded as a cross sectional study.

97. Overmeer T., Boersma K., Main CJ. & Linton SJ. (2009) Do physical therapists change their beliefs, attitudes, knowledge, skills and behaviour after a biopsychosocially orientated university course? Journal of Evaluation in Clinical Practice 15:724-732

Excluded as no assessment of healthcare practitioner behaviour change, healthcare system change or effect on patient outcomes.

98. Overmeer T., Boersma K., Denison E. & Linton SJ. (2011) Does teaching physical therapists to deliver a biopsychosocial treatment program result in better patient outcomes? A randomised controlled trial. Physical Therapy 91(5):804-819

Excluded as not specific to non-specific low back pain.

99. Parkin-Smith GF., Norman IJ., Briggs E., Angier E., Wood TG. & Brantingham JW. (2012) A structured protocol of evidence-based conservative care compared with usual care for acute nonspecific low back pain: a randomized clinical trial. Archives of Physical Medicine & Rehabilitation 93(1):11-20.

Excluded as no assessment of healthcare practitioner behaviour change, healthcare system change or effect on patient outcomes.

100. Patel S., Brown S., Friede T., Griffiths F., Lord J., Ngunjiri A., Thistlethwaite J., Tysall C., Woolvine M. & Underwood M. (2011) Study protocol: Improving patient choice in treating low back pain (IMPACT - LBP): A randomised controlled trial of a decision support package for use in physical therapy. BMC Musculoskeletal Disorders 12:52.

Excluded as protocol paper only.

101. Pinnington MA., Miller J. & Stanley I. (2004) An evaluation of prompt access to physiotherapy in the management of low back pain in primary care. Family Practice 21(4):372-80.

Duplicate see 88.

102. Rantonen J., Luoto S., Vehtari A., Hupli M., Karppinen J., Malmivaara A. & Taimela S. (2012) The effectiveness of two active interventions compared to self-care advice in employees with non-acute low back symptoms: a randomised, controlled trial with a 4-year follow-up in the occupational health setting. Occupational and Environmental Medicine 69(1):12-20.

Excluded as no assessment of healthcare practitioner behaviour change, healthcare system change or effect on patient outcomes.

103. Rao JK., Kroenke K., Mihaliak KA., Eckert GJ. & Weinberger M. (2002) Can guidelines impact the ordering of magnetic resonance imaging studies by primary care providers for low back pain? American Journal of Managed Care 8(1):27-35.

Excluded as retrospective cohort study.

104. Roberts L., Little P., Chapman J., Cantrell T., Pickering R. & Langridge J. (2002) The back home trial: general practitioner-supported leaflets may change back pain behavior. Spine 27(17):1821-8.

Excluded as no assessment of healthcare practitioner behaviour change, healthcare system change or effect on patient outcomes.

105. Robling MR., Houston HL., Kinnersley P., Hourihan MD., Cohen DR., Hale J. & Hood K. (2002) General practitioners’ use of magnetic resonance imaging: an open randomized trial comparing telephone and written requests and an open randomized controlled trial of different methods of local guideline dissemination. Clinical Radiology 57(5):402–7.

Excluded as results mix knee and lumbar spine data.

106. Rogerson MD., Gatchel RJ. & Bierner SM. (2010) A cost utility analysis of interdisciplinary early intervention versus treatment as usual for high-risk acute low back pain patients. Pain Practice 10(5):382-95.

Excluded as no assessment of healthcare practitioner behaviour change, healthcare system change or effect on patient outcomes.

107. Rosemann T., Joos S., Koerner T., Heiderhoff M., Laux G. & Szecsenyi J. (2006) Use of a patient information leaflet to influence patient decisions regarding mode of administration of NSAID medications in case of acute low back pain. European Spine Journal 15(11):1737-1741.

Excluded as no assessment of healthcare practitioner behaviour change, healthcare system change or effect on patient outcomes.

108. Rossignol M., Abenhaim L., Seguin P., Neveu A., Collet JP., Ducruet T. & Shapiro S. (2000) Coordination of primary health care for back pain. A randomized controlled trial. 25(2):251-259.

Excluded as mixed thoracic and lumbar spine conditions.

109. Rutten GM, Harting J, Rutten ST, Bekkering GE, Kremers SP. Measuring physiotherapists' guideline adherence by means of clinical vignettes: a validation study. Journal of evaluation in clinical practice 2006;12(5):491-500.

Excluded as a validation study.

110. Rutten G., Kremers S., Rutten S. & Harting J. (2009) A theory-based cross-sectional survey demonstrated the important role of awareness in guideline implementation. Journal of Clinical Epidemiology 62:167-176

Excluded as a cross-sectional survey.

111. Rutten GM., Degen S., Hendriks EJ., Braspenning JC., Harting J. & Oostendrop. (2010) Adherence to clinical practice guidelines for low back pain in physical therapy: Do patients benefit? Physical Therapy 90(8):1111-1122

Excluded as an observational prospective cohort study.

112. Sahin N., Albayrak I., Durmus B. & Ugurlu H. (2011) Effectiveness of back school for treatment of pain and functional disability in patients with chronic low back pain: A randomized controlled trial. Journal of Rehabilitation Medicine 43(3):224-9.

Excluded as no assessment of healthcare practitioner behaviour change, healthcare system change or effect on patient outcomes.

113. Salisbury C., Foster NE., Bishop A., Calnan M., Coast J., Hall J., Hay E., Hollinghurst S., Hopper C., Grove S., Kaur S. & Montgomery A. (2009) 'PhysioDirect' telephone assessment and advice services for physiotherapy: protocol for a pragmatic randomised controlled trial. BMC Health Services Research 9:136.

Excluded as protocol only.

114. Scheel IB., Birger K., Herrin J. & Oxman AD. (2002) A call for action: A randomized controlled trial of two strategies to implement active sick leave for patients with low back pain. Spine 27(6):561-66.

Excluded as mixed non-specific low back pain conditions as well as specific conditions.

115. Scheel IB., Hagen KB., Herrin J., Carling C. & Oxman AD. (2002) Blind faith? The effects of promoting active sick leave for back pain patients: a cluster-randomized controlled trial. Spine 27(23):2734-40.

Excluded as mixed non-specific low back pain conditions as well as specific conditions.

116. Schulz PJ., Rubinell S. & Hartung U. (2007) An internet-based approach to enhance self-management of chronic low back pain in the Italian-speaking population of Switzerland: results from a pilot study. International Journal of Public Health 52(5):286-94.

Excluded as no assessment of healthcare practitioner behaviour change, healthcare system change or effect on patient outcomes.

117. Schulz PJ., Rubinelli S., Zufferey MC. & Hartung U. (2010) Coping With Chronic Lower Back Pain: Designing and Testing the Online Tool ONESELF. Journal of Computer-Mediated Communication 15(4):625-45.

Excluded as no assessment of healthcare practitioner behaviour change, healthcare system change or effect on patient outcomes.

118. Schuring M., Burdorf A., Voorham AJ., der Weduwe K. & Mackenbach JP. (2009) Effectiveness of a health promotion programme for long-term unemployed subjects with health problems: a randomised controlled trial. Journal of Epidemiology and Community Health 63(11):893-99.

Excluded as not non-specific low back pain.

119. Schutgens CAE., Schuring M., Voorham TAJ. & Burdorf A. (2009) Changes in physical health among participants in a multidisciplinary health programme for long-term unemployed persons. BMC Public Health 9:197.

Excluded as no assessment of healthcare practitioner behaviour change, healthcare system change or effect on patient outcomes.

120. Selfridge NJ. (2010) Integrated Care for Low Back Pain: Focus on Gain. Alternative Medicine Alert 13(6):67-68.

Excluded as is a commentary on another study only.

121. Shekelle PG., Kravitz RL., Beart J., Marger M., Wang MM. & Lee M. (2000) Are nonspecific practice guidelines potentially harmful? A randomized comparison of the effect of nonspecific versus specific guidelines on physician decision making. Health Services Research 34(7):1429-48.

Excluded as belief change assessed but not actual behaviour change.

122. Slater H, Davies SJ, Parsons R, Quintner JL, Schug SA. A policy-into-practice intervention to increase the uptake of evidence-based management of low back pain in primary care: A prospective cohort study. PLoS ONE 2012;7(5).

Excluded as belief change and intended behaviours measured only.

123. Sowden G., Hill JC., Konstantinou K., Khanna M., Main CJ., Salmon P., Somerville S., Wathall S. & Foster NE. (2012) Team IMBS. Targeted treatment in primary care for low back pain: the treatment system and clinical training programmes used in the IMPaCT Back study. Family Practice 29(1):50-62.

Excluded as a protocol only.

124. Taylor VM., Goldberg HI., Deyo RA., Cooper S., Leek M., Norduglen LL., Spunt B. & Conrad D. (1996) Modifying community practice styles: the Back Pain Outcome Assessment Team information dissemination effort. Journal of Continuing Education in the Health Professions 16(4):203-214

Excluded as a description of the implementation process but no results provided.

125. Tracey NG., Martin JB., McKinstry CS. & Mathew BM. (1994) Guidelines for lumbar spine radiography in acute low back pain: effect of implementation in an accident and emergency department. Ulster Medical Journal 63(1):12-7.

Excluded as an uncontrolled before and after study.

126. van der Roer N., van Tulder M., Barendse J, Knol D, van Mechelen W, de Vet H. (2008) Intensive group training protocol versus guideline physiotherapy for patients with chronic low back pain: a randomised controlled trial. European Spine Journal 17(9):1193-1200.

Excluded as no assessment of healthcare practitioner behaviour change, healthcare system change or effect on patient outcomes.

127. van der Roer N., van Tulder M, van Mechelen W. & de Vet H. (2008) Economic evaluation of an intensive group training protocol compared with usual care physiotherapy in patients with chronic low back pain. Spine 33(4):445-51.

Excluded as aim of study was not to implement change.

128. van Tulder M., van Mechelen W. & de Vet H. (2008) Economic evaluation of an intensive group training protocol compared with usual care physiotherapy in patients with chronic low back pain. Spine 33(4):445-51.

Excluded as duplicate of 116.

129. Vermeulen SJ., Anema JR., Schellart AJM., van Mechelen W. & van der Beek AJ. (2009) Intervention mapping for development of a participatory return-to-work intervention for temporary agency workers and unemployed workers sick-listed due to musculoskeletal disorders. BMC Public Health 9:216.

Excluded as no assessment of healthcare practitioner behaviour change, healthcare system change or effect on patient outcomes.

130. Vermeulen SJ., Anema JR., Schellart AJM., van Mechelen W. & van der Beek AJ. (2010) Cost-effectiveness of a participatory return-to-work intervention for temporary agency workers and unemployed workers sick-listed due to musculoskeletal disorders: design of a randomised controlled trial. BMC Musculoskeletal Disorders 11:60.

Excluded as no assessment of healthcare practitioner behaviour change, healthcare system change or effect on patient outcomes.

131. Von M., Balderson BH., Saunders K., Miglioretti DL., Lin EH., Berry S., Moore JE. & Turner JA. (2005) A trial of an activating intervention for chronic back pain in primary care and physical therapy settings. Pain 113(3):323-30.

Excluded as no assessment of healthcare practitioner behaviour change, healthcare system change or effect on patient outcomes.

132. Von M., Moore JE., Lorig K., Cherkin DC., Saunders K., Gonzalez VM., Laurent D., Rutter C. & Comite F. (1998) A randomized trial of a lay person-led self-management group intervention for back pain patients in primary care. Spine 23(23):2608-15.

Excluded as no assessment of healthcare practitioner behaviour change, healthcare system change or effect on patient outcomes.

133. Waddell G., O'Connor M., Boorman S. & Torsney B. (2007) Working Backs Scotland: a public and professional health education campaign for back pain. Spine 32(19):2139-43.

Excluded as time interrupted series study but with only one measure pre-intervention.

134. Werner EL., Gross DP., Lie SA. & Ihlebaek C. (2008) Healthcare provider back pain beliefs unaffected by a media campaign. Scandinavian Journal of Primary Health Care 26(1):50-6.

Excluded as belief change assessed but not actual behaviour change.

135. Werner EL., Ihlebaek C., Laerum E., Wormgoor ME. & Indahl A. (2008) Low back pain media campaign: no effect on sickness behaviour. Patient Education & Counseling 71(2):198-203.

Excluded as no assessment of healthcare practitioner behaviour change, healthcare system change or effect on patient outcomes.

136. Zwerver F., Schellart AJM., Knol DL., Anema JR. & van der Beek AJ. (2011) An implementation strategy to improve the guideline adherence of insurance physicians: an experiment in a controlled setting. Implementation Science 6:131.

Excluded as not non-specific low back pain.
